# Supplementary material for: Genomic insights into ancestry and infectious disease in 17th-century colonial Brazil
Source: bioRxiv. 2025 Dec 11:2025.12.10.693243. Preprint. [Version 1] doi: 10.64898/2025.12.10.693243 (PMC12710828; doi:10.64898/2025.12.10.693243)
Supplement: Supplement 1 [file media-1.pdf]

Supplementary material

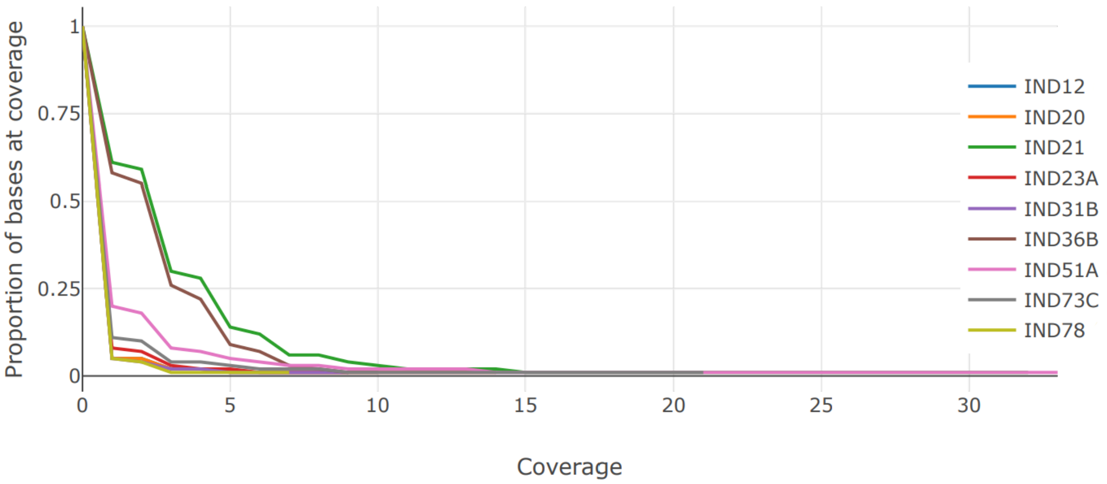

Figure S1: Coverage distribution across the nine Pilar individuals. The plot shows the proportion of genomic bases observed at each coverage level, providing an overview of sequencing depth and data completeness for all samples. Coverage values are based on genome-wide estimates.

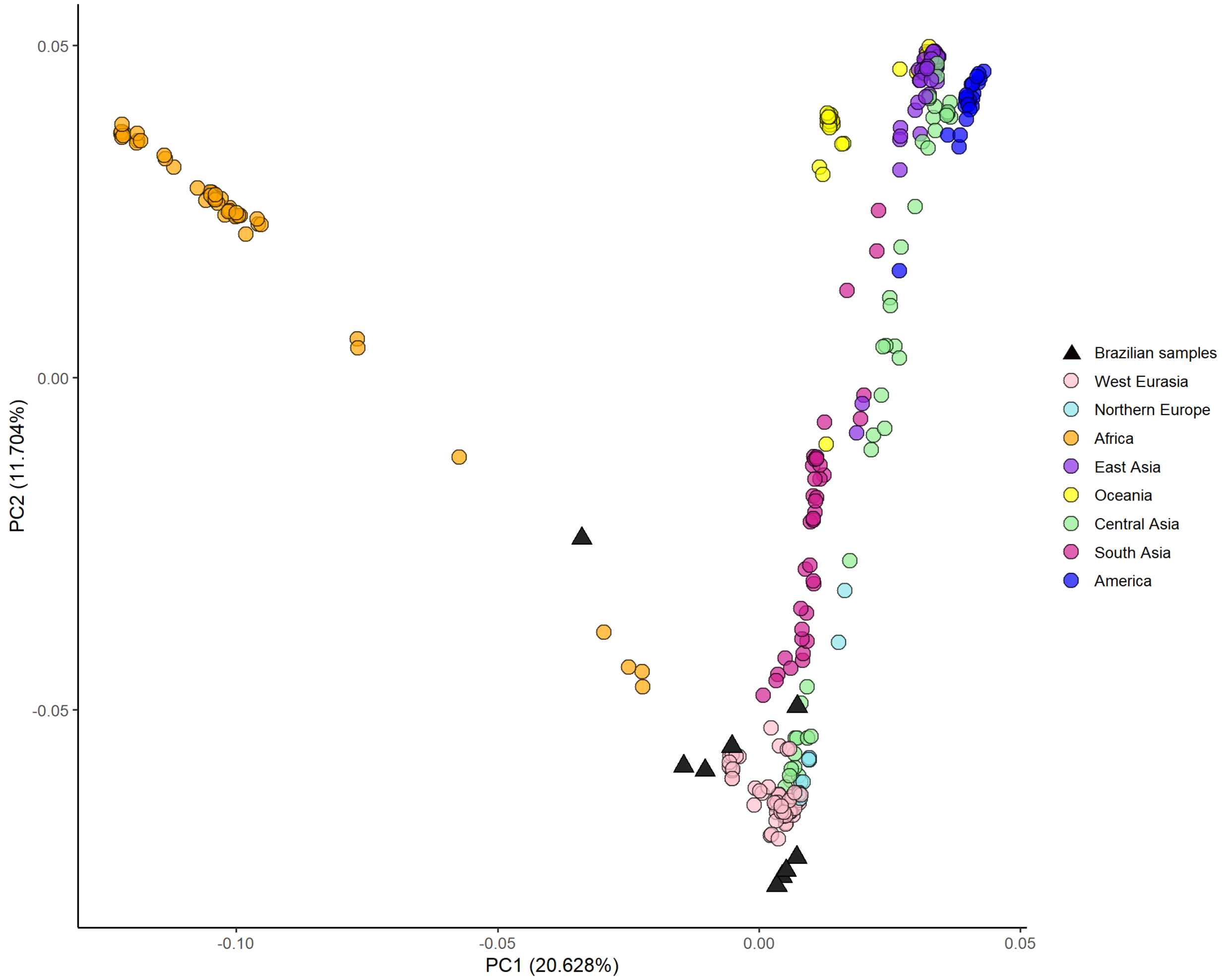

Figure S2: PCA for all nine Pilar individuals (black triangles), projected onto present-day reference populations from the SGDP dataset. Lower-coverage genomes display greater projection variability. Reference population colors correspond to major geographic regions.

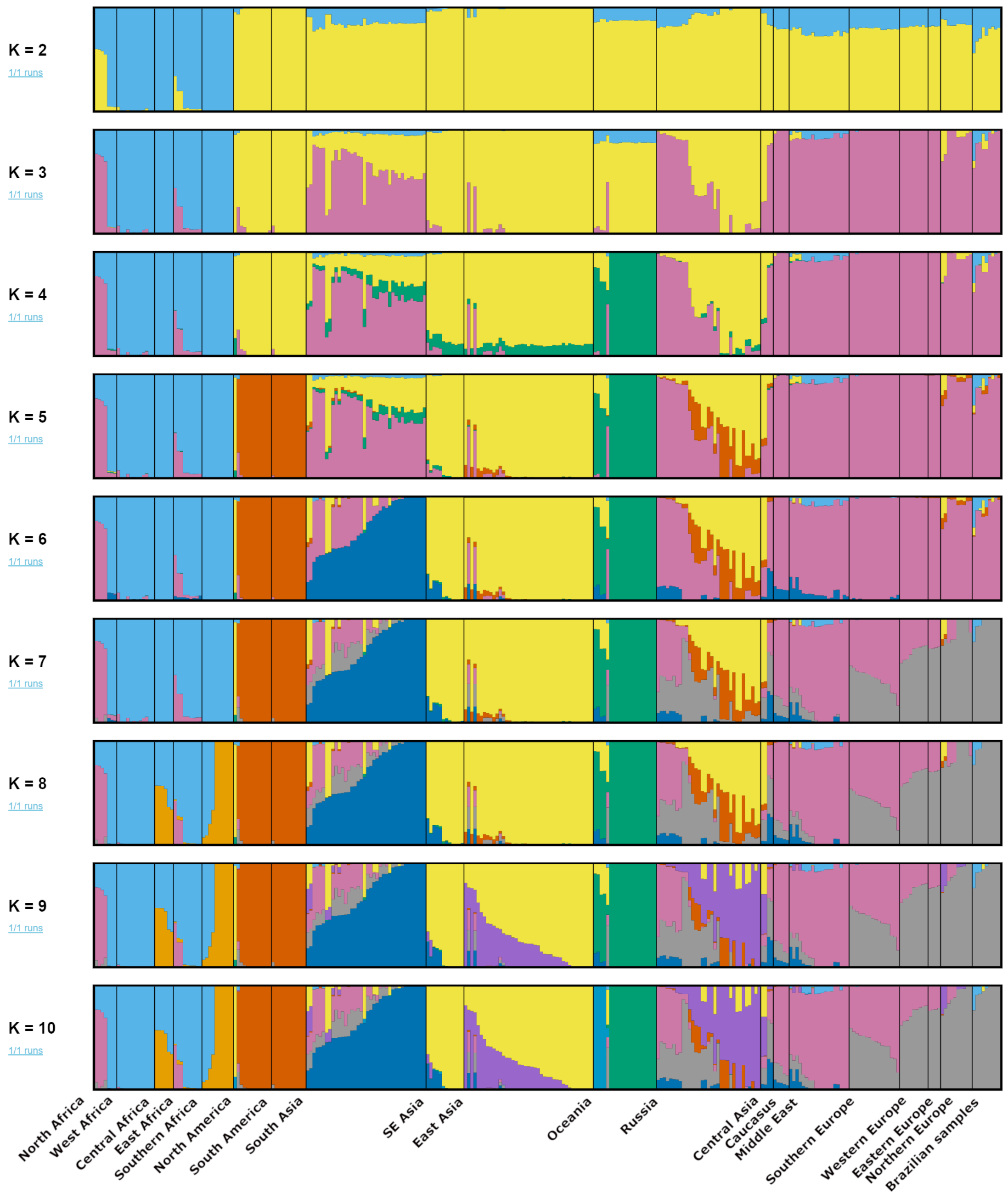

Figure S3: ADMIXTURE analysis results for $K = 2$ through $K = 10$, including all nine samples. Each vertical bar corresponds to one individual and is subdivided into ancestry components, with color segment height indicating the proportion of each component. The Pilar individuals are shown in the final segment of the plot.

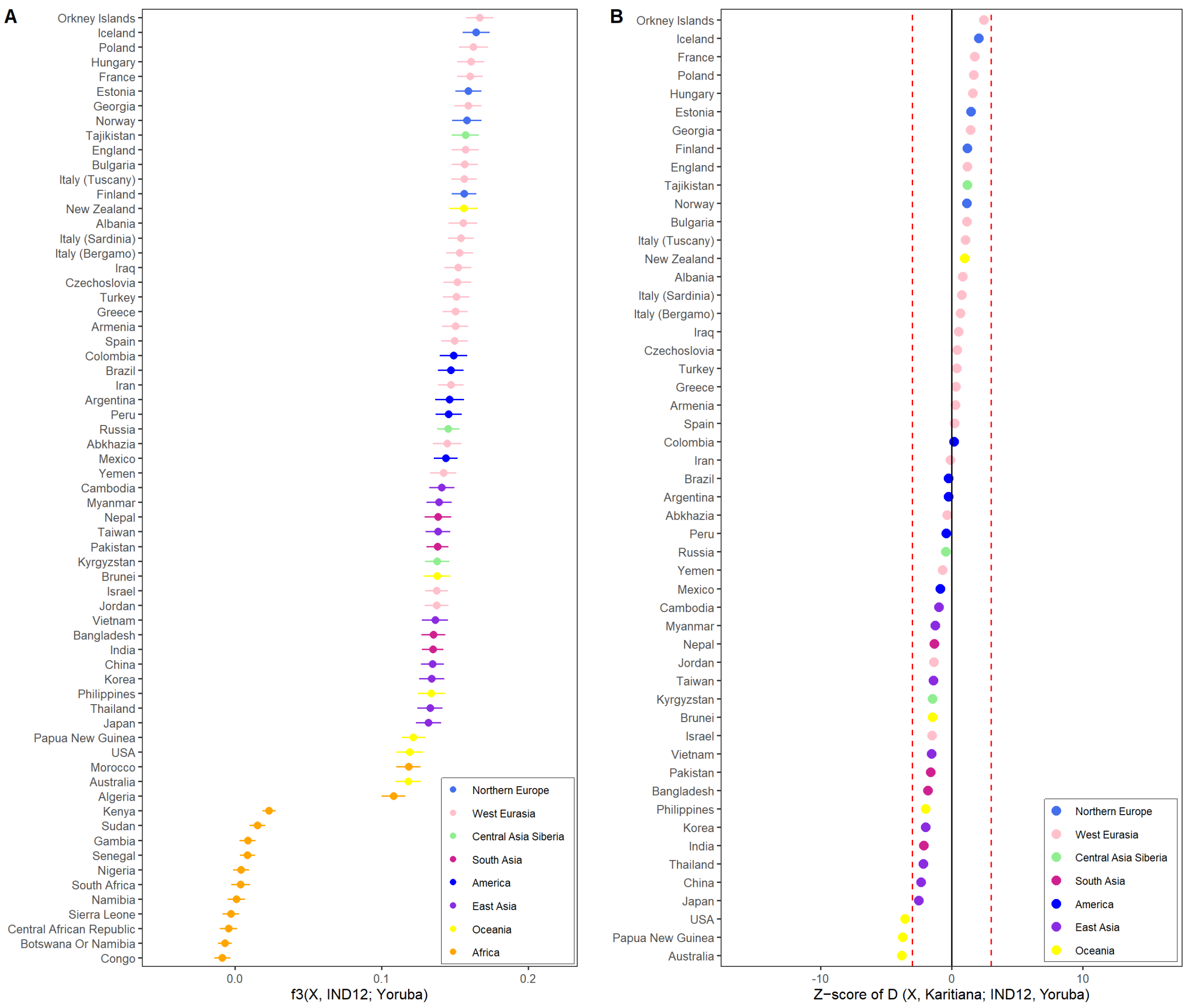

Figure S4: Outgroup f_3 and D-statistic results for IND12. (A) Outgroup f_3 statistics for IND12, computed in the form $f_3(X, \text{IND12}; \text{Yoruba})$ and ranked by decreasing affinity across comparison populations. (B) D-statistics, computed as $D(X, \text{Karitiana}; \text{IND12}, \text{Yoruba})$ and ranked by decreasing Z-score. Positive values indicate excess allele sharing between IND12 and population X relative to Karitiana, while negative values indicate the reverse. Vertical dashed lines at $Z = \pm 3$ mark the conventional thresholds used to assess significance.

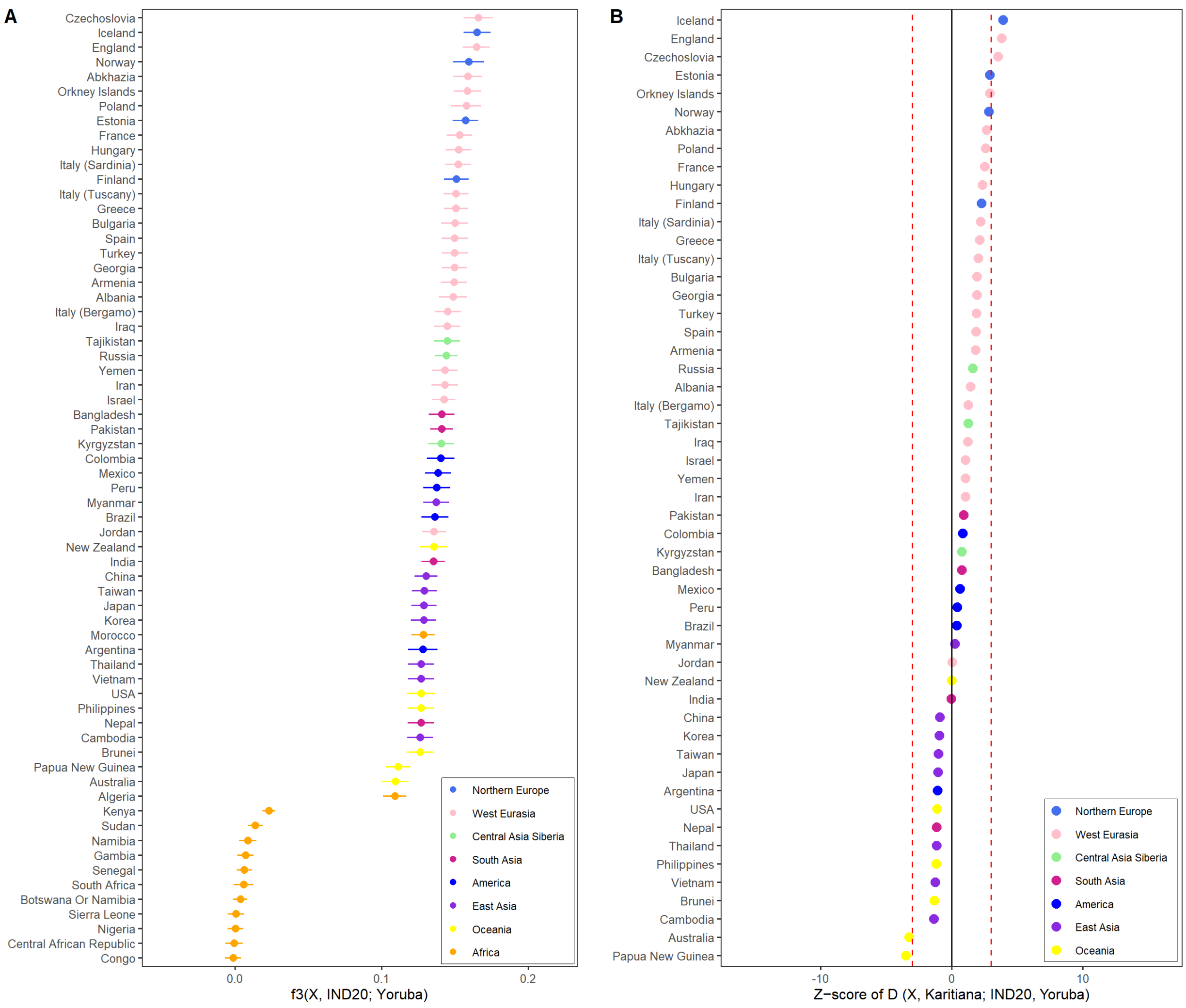

Figure S5: Outgroup f_3 and D-statistic results for IND20. (A) Outgroup f_3 statistics for IND20, computed in the form $f_3(X, \text{IND20}; \text{Yoruba})$ and ranked by decreasing affinity across comparison populations. (B) D-statistics, computed as $D(X, \text{Karitiana}; \text{IND20}, \text{Yoruba})$ and ranked by decreasing Z-score. Positive values indicate excess allele sharing between IND20 and population X relative to Karitiana, while negative values indicate the reverse. Vertical dashed lines at $Z = \pm 3$ mark the conventional thresholds used to assess significance.

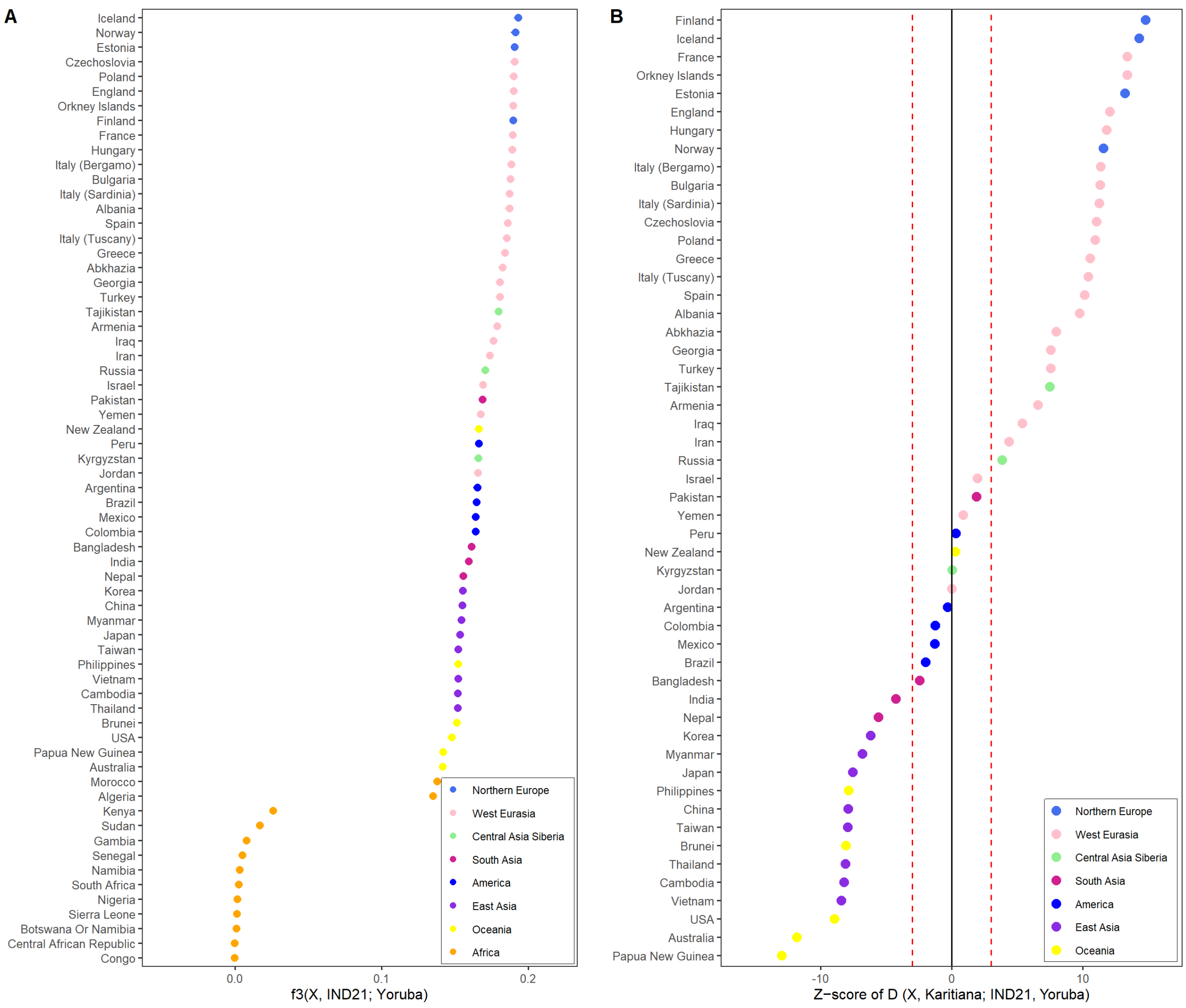

Figure S6: Outgroup f_3 and D-statistic results for IND21. (A) Outgroup f_3 statistics for IND21, computed in the form $f_3(X, \text{IND21}; \text{Yoruba})$ and ranked by decreasing affinity across comparison populations. (B) D-statistics, computed as $D(X, \text{Karitiana}; \text{IND21}, \text{Yoruba})$ and ranked by decreasing Z-score. Positive values indicate excess allele sharing between IND21 and population X relative to Karitiana, while negative values indicate the reverse. Vertical dashed lines at $Z = \pm 3$ mark the conventional thresholds used to assess significance.

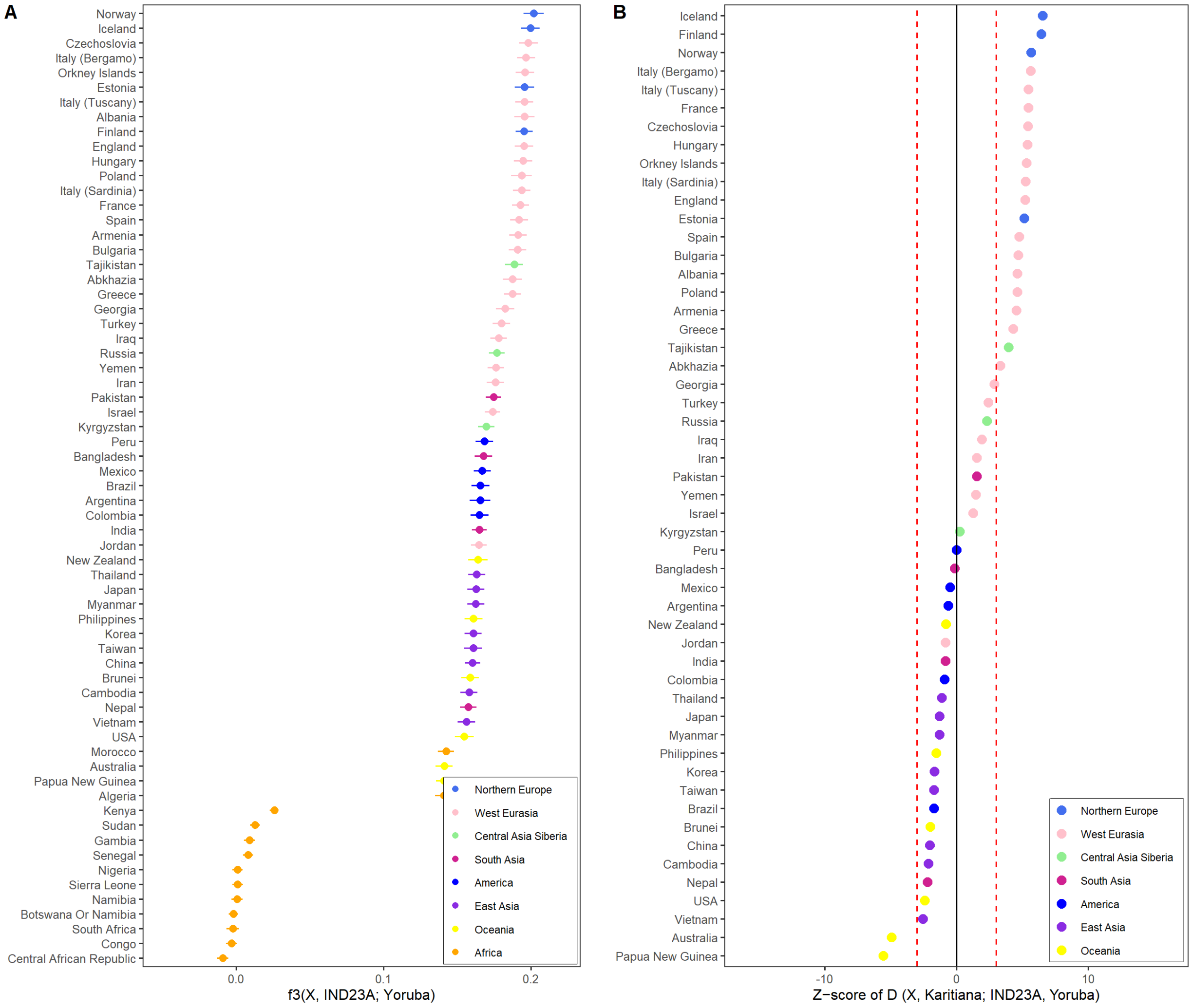

Figure S7: Outgroup f_3 and D-statistic results for IND23A. (A) Outgroup f_3 statistics for IND23A, computed in the form $f_3(X, \mathrm{IND23A}; \mathrm{Yoruba})$ and ranked by decreasing affinity across comparison populations. (B) D-statistics, computed as $D(X, \mathrm{Karitiana}; \mathrm{IND23A}, \mathrm{Yoruba})$ and ranked by decreasing Z-score. Positive values indicate excess allele sharing between IND23A and population X relative to Karitiana, while negative values indicate the reverse. Vertical dashed lines at $Z = \pm 3$ mark the conventional thresholds used to assess significance.

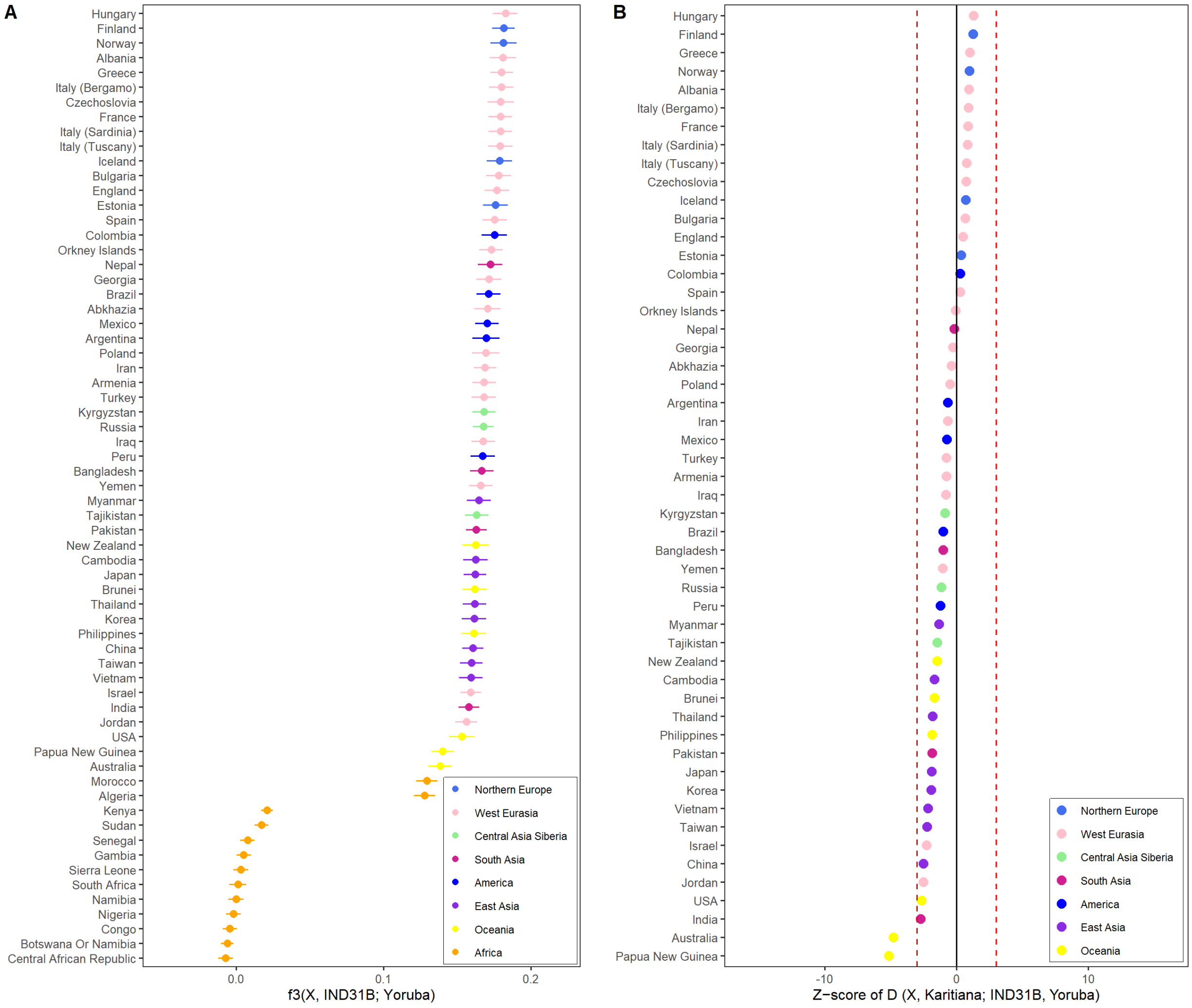

Figure S8: Outgroup f_3 and D-statistic results for IND31B. (A) Outgroup f_3 statistics for IND31B, computed in the form $f_3(X, \text{IND31B}; \text{Yoruba})$ and ranked by decreasing affinity across comparison populations. (B) D-statistics, computed as $D(X, \text{Karitiana}; \text{IND31B}, \text{Yoruba})$ and ranked by decreasing Z-score. Positive values indicate excess allele sharing between IND31B and population X relative to Karitiana, while negative values indicate the reverse. Vertical dashed lines at $Z = \pm 3$ mark the conventional thresholds used to assess significance.

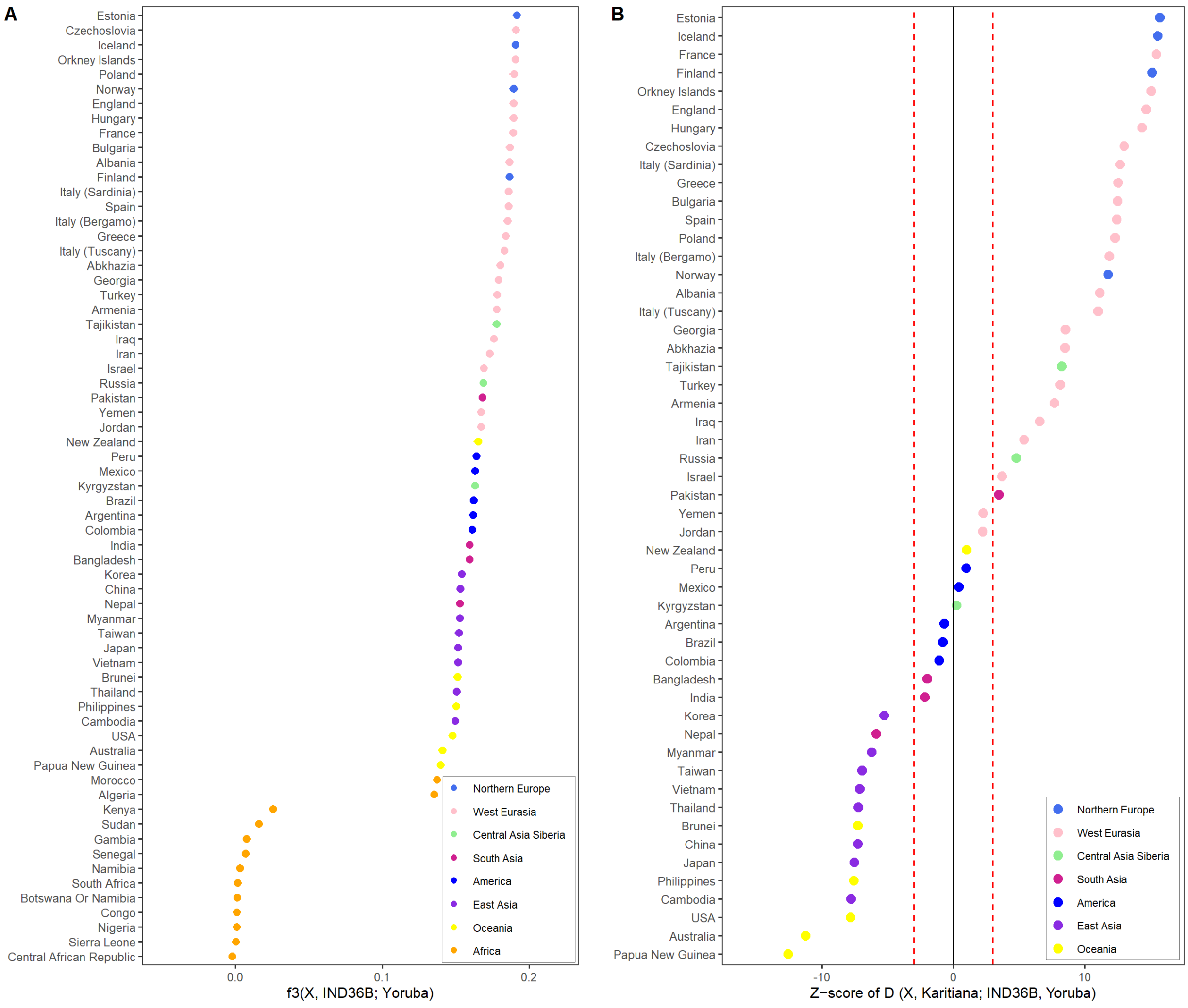

Figure S9: Outgroup f_3 and D-statistic results for IND36B. (A) Outgroup f_3 statistics for IND36B, computed in the form $f_3(X, \text{IND36B}; \text{Yoruba})$ and ranked by decreasing affinity across comparison populations. (B) D-statistics, computed as $D(X, \text{Karitiana}; \text{IND36B}, \text{Yoruba})$ and ranked by decreasing Z-score. Positive values indicate excess allele sharing between IND36B and population X relative to Karitiana, while negative values indicate the reverse. Vertical dashed lines at $Z = \pm 3$ mark the conventional thresholds used to assess significance.

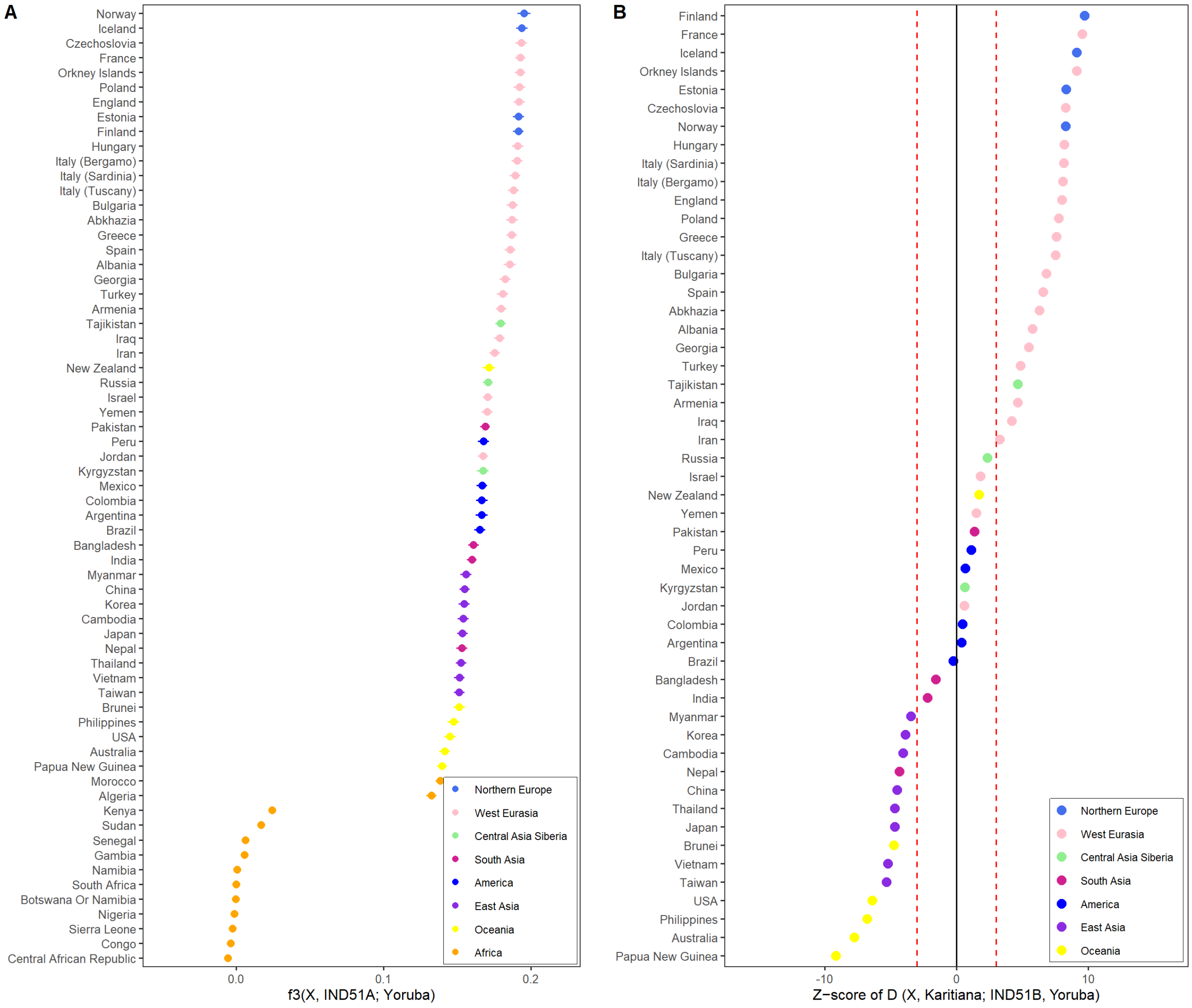

Figure S10: Outgroup f_3 and D-statistic results for IND51A. (A) Outgroup f_3 statistics for IND51A, computed in the form $f_3(X, \text{IND51A}; \text{Yoruba})$ and ranked by decreasing affinity across comparison populations. (B) D-statistics, computed as $D(X, \text{Karitiana}; \text{IND51A}, \text{Yoruba})$ and ranked by decreasing Z-score. Positive values indicate excess allele sharing between IND51A and population X relative to Karitiana, while negative values indicate the reverse. Vertical dashed lines at $Z = \pm 3$ mark the conventional thresholds used to assess significance.

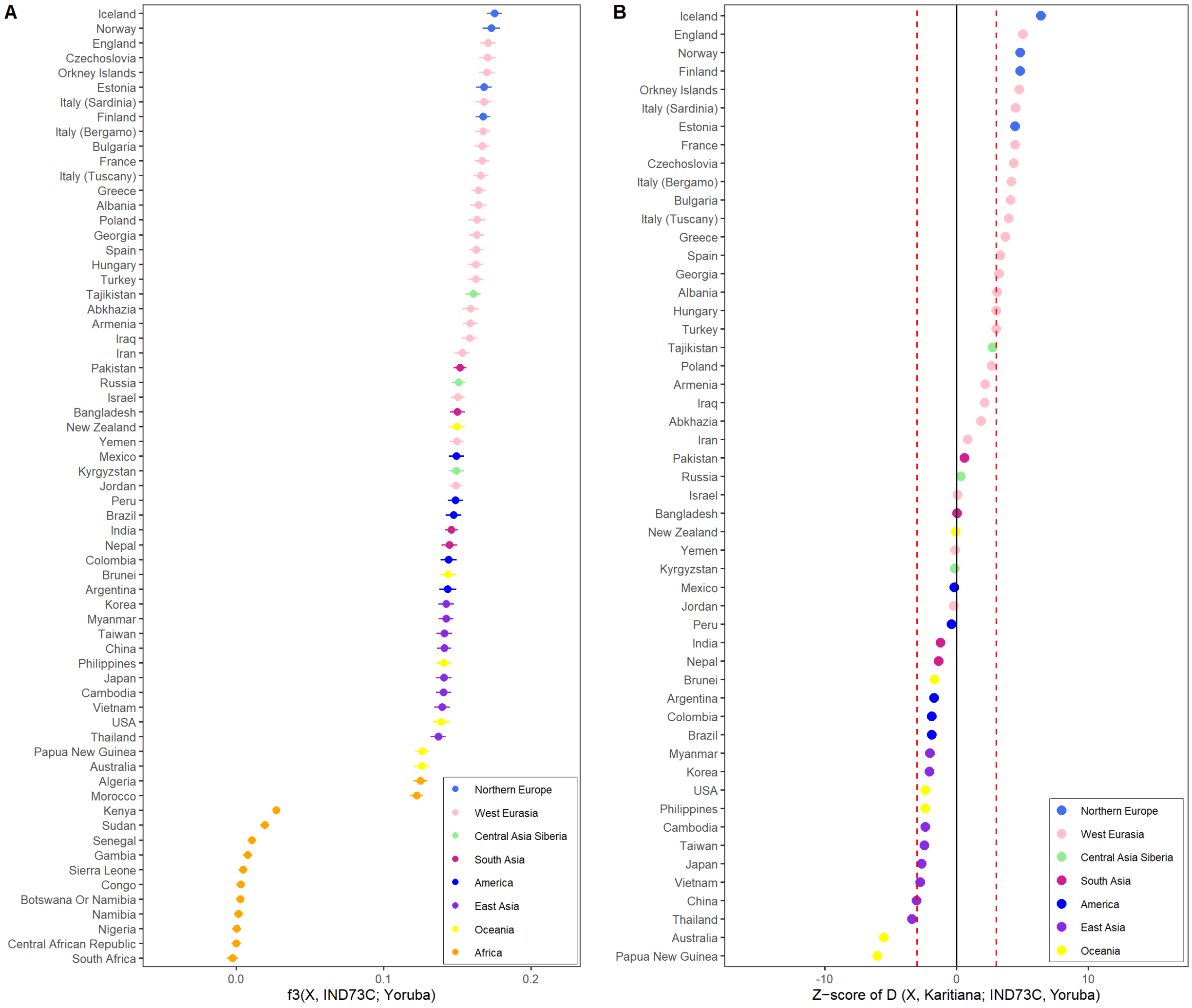

Figure S11: Outgroup f_3 and D-statistic results for IND73C. (A) Outgroup f_3 statistics for IND73C, computed in the form $f_3(X, \text{IND73C}; \text{Yoruba})$ and ranked by decreasing affinity across comparison populations. (B) D-statistics, computed as $D(X, \text{Karitiana}; \text{IND73C}, \text{Yoruba})$ and ranked by decreasing Z-score. Positive values indicate excess allele sharing between IND73C and population X relative to Karitiana, while negative values indicate the reverse. Vertical dashed lines at $Z = \pm 3$ mark the conventional thresholds used to assess significance.

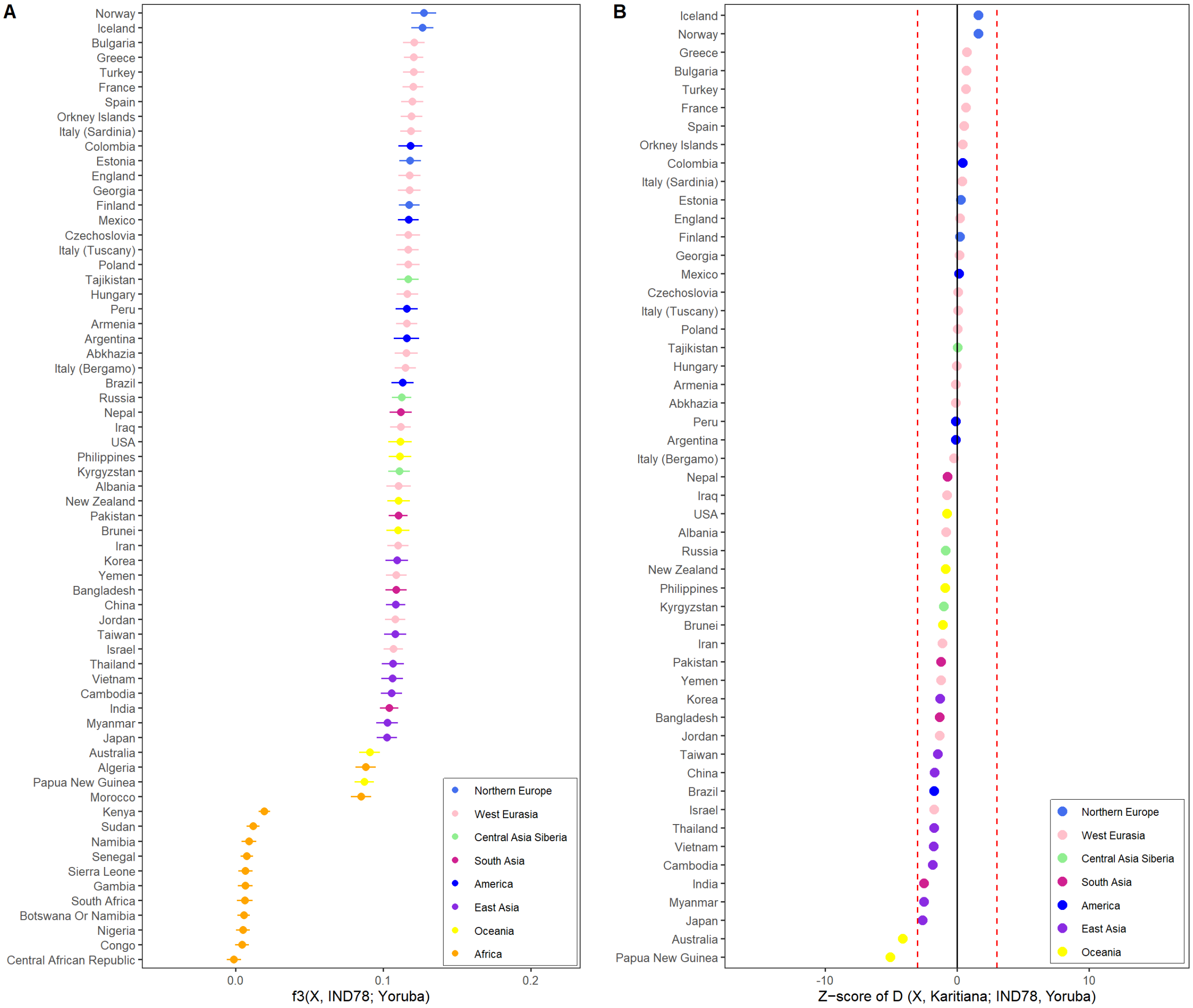

Figure S12: Outgroup f_3 and D-statistic results for IND78. (A) Outgroup f_3 statistics for IND78, computed in the form $f_3(X, \text{IND78}; \text{Yoruba})$ and ranked by decreasing affinity across comparison populations. (B) D-statistics, computed as $D(X, \text{Karitiana}; \text{IND78}, \text{Yoruba})$ and ranked by decreasing Z-score. Positive values indicate excess allele sharing between IND78 and population X relative to Karitiana, while negative values indicate the reverse. Vertical dashed lines at $Z = \pm 3$ mark the conventional thresholds used to assess significance.

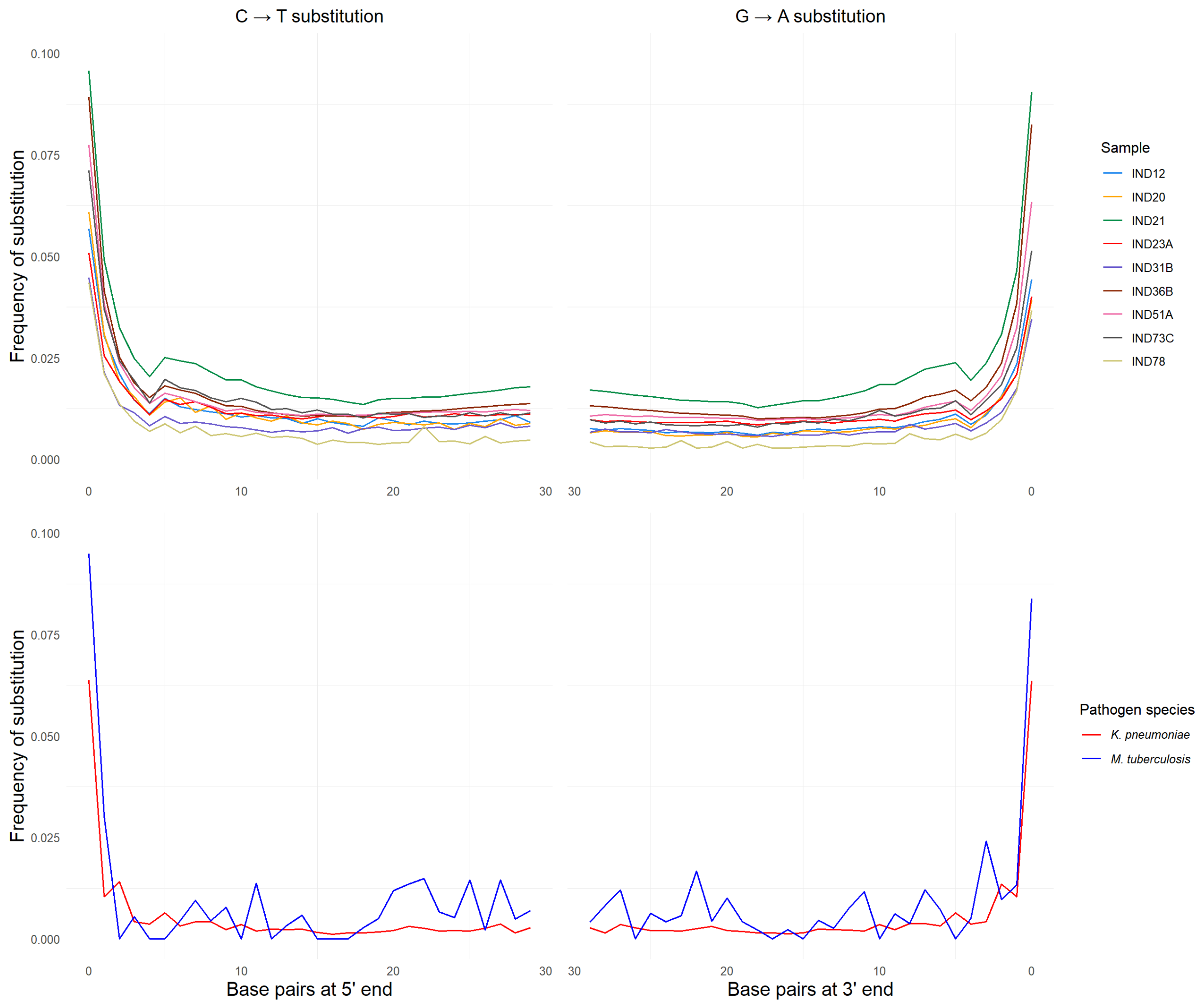

Figure S13: Postmortem damage profiles inferred using PMDtools. Left panels show 5′-end C→T substitution frequencies, and right panels show 3′-end G→A substitution frequencies across read positions. Damage patterns are displayed for the nine Pilar individuals (top) and for *K. pneumoniae* and *M. tuberculosis* reads recovered from IND21, the individual yielding the highest number of pathogen-derived sequences (bottom). Elevated terminal deamination rates in both human and microbial DNA are consistent with authentic ancient DNA. The *M. tuberculosis* curve exhibits greater variance due to its substantially lower read depth relative to *K. pneumoniae*.

Table S1: Uniparental haplogroups and autosomal coverage metrics for the nine Pilar individuals.

Sample	mtDNA haplogroup	Y haplogroup	Mean depth	Mean coverage
IND12	H1u	P-P337	0.321	0.054
IND20	W1c	R-Z159	0.347	0.051
IND21	H1b	R-S23634	1.578	0.486
IND23A	H70	A-Y156117	0.450	0.084
IND31B	H26a1	R-YP5802	0.264	0.051
IND36B	H2a2	R-Y130457	2.028	0.590
IND51A	U2e2a1c	R-L52	1.167	0.201
IND73C	I1a1	R-Y3159	0.771	0.115
IND78	H3+152	R-M269	0.295	0.047
